# Impact of an intervention for osteoarthritis based on exercise and education on metabolic health: a register-based study using the SOAD cohort

**DOI:** 10.1136/rmdopen-2024-005133

**Published:** 2025-02-26

**Authors:** Simone Battista, Filippo Recenti, Ali Kiadaliri, Stefan Lohmander, Thérése Jönsson, Allan Abbott, Johanna Vinblad, Ola Rolfson, Martin Englund, Andrea Dell’Isola

**Affiliations:** 1 School of Health and Society, Centre for Human Movement and Rehabilitation, University of Salford, Salford, UK; 2 Department of Clinical Sciences Lund, Clinical Epidemiology Unit, Orthopaedics, Lund University, Lund, Sweden; 3 Department of Neurosciences, Rehabilitation, Ophthalmology, Genetics, Maternal and Child Health, University of Genova, Genova, Italy; 4 Faculty of Medicine, Department of Clinical Sciences Lund, Orthopaedics, Lund University, Lund, Sweden; 5 Faculty of Medicine, Department of Health Sciences, Lund University, Lund, Sweden; 6 Orthopaedics, Skane University Hospital, Lund, Sweden; 7 Department of Health, Medicine and Caring Sciences, Unit of Physiotherapy, Linköping University, Linkoping, Sweden; 8 Department of Orthopaedics, Linköping University Hospital, Linköping, Sweden; 9 Centre of Registers Västra Götaland, The Swedish Hip Arthroplasty Register, Goteborg, Sweden; 10 Department of Orthopaedics, Institute of Clinical Sciences, Sahlgrenska Academy, University of Gothenburg, Goteborg, Sweden

**Keywords:** Osteoarthritis, Physical Therapy Modalities, Health services research

## Abstract

**Objective:**

This study evaluated the effects of a 6-week osteoarthritis (OA) exercise and education intervention on metabolic health markers, including blood pressure (BP), glycated haemoglobin (HbA1c), high-density lipoprotein (HDL), cholesterol levels and weight in individuals with both OA and diabetes.

**Methods:**

Data originated from the Swedish Osteoarthritis and Diabetes cohort, which is composed of the Swedish Osteoarthritis Register (SOAR) and National Diabetes Register. We included individuals diagnosed with OA and diabetes who underwent the intervention between January 2008 and December 2019, matched with controls with diabetes who did not based on birth year, sex, OA site (hip/knee) and OA diagnosis year. Outcomes included BP, HbA1c, HDL, total cholesterol levels and weight measured up to 3 years before and after SOAR enrolment. Statistical analyses used two-way fixed-effect models.

**Results:**

The study included 4571 individuals with OA and diabetes (mean age: 69.5, SD: 7.8; women: 52.7%; knee OA: 71.2%) and 7925 controls. The intervention group showed a systolic BP decrease of approximately 1.0 mm Hg at 6 and 12 months compared with the control group. HDL levels increased by about 0.02 mmol/L at 12, 18 and 24 months. Weight decreased by approximately 0.5 kg at 6, 18 and 30 months. HbA1c levels increased by approximately 0.5 mmol/mol at 6 months. No essential differences were found in the total cholesterol levels.

**Conclusion:**

An OA exercise and education intervention designed following OA clinical practice guidelines led to small and unlikely clinically relevant improvements in metabolic health markers in individuals with OA and diabetes.

**WHAT IS ALREADY KNOWN ON THIS TOPIC**

- Osteoarthritis (OA) and diabetes frequently coexist. Exercise is widely recommended for OA and diabetes to improve joint health and metabolic outcomes. However, adherence to exercise recommendations is challenging for those with both conditions, and limited evidence exists on the impact of OA first-line interventions on metabolic health in this population.

**WHAT THIS STUDY ADDS**

- This study shows that a 6-week self-management education and exercise intervention for OA designed following clinical practice guidelines led to small and unclear clinical significance improvements in the metabolic health of people with OA and diabetes.

**HOW THIS STUDY MIGHT AFFECT RESEARCH, PRACTICE OR POLICY**

- The findings suggest that first-line OA management based on clinical practice guidelines provides small metabolic benefits for individuals with diabetes and OA. Future interventions and clinical practice guidelines may need to be more tailored to people with comorbid OA and diabetes.

## Introduction

 Osteoarthritis (OA) and diabetes stand as two of the most prevalent long-term conditions globally, contributing significantly to disability rates worldwide, with their prevalence steadily increasing.^1 2^ These conditions often occur together, with 30% of people with OA also having diabetes, compared with 13% in the general population.^3^ Research indicates that diabetes is associated with heightened pain intensity and decreased mobility in people with OA.^4 5^ In the management of OA and diabetes, exercise is considered a first-line intervention^6 7^ for its supposed ability to reduce joint pain and systemic inflammation^8^ while improving physical function^9 10^ and metabolic control—including blood pressure (BP), glycated haemoglobin (HbA1c), high-density lipoprotein (HDL), cholesterol levels and body weight.^711^,^14^ Guidelines for both conditions recommend physical activity and exercise based on the WHO or American College of Sports Medicine guidelines, which include a comprehensive combination of cardiorespiratory, resistance, flexibility and neuromotor exercise training.^6 15^ Moreover, education on self-management strategies is recommended in OA to explain the importance of maintaining adequate exercise levels and symptom management.^6^

However, people with OA and diabetes struggle with reaching recommended levels of exercise.^16 17^ The coexistence of these two conditions further exacerbates the difficulty of engaging in exercise, as the joint pain caused by OA may represent a barrier to engaging in exercise programmes designed to improve metabolic health that do not account for the presence of OA.^18^ First-line management programmes for OA are based on exercise and an education component on self-management that often promotes an active lifestyle.^6^ Therefore, such programmes have the potential to improve also the metabolic health of the participant, but evidence is lacking.

This project sought to investigate the impact of the 6-week self-management education and exercise intervention for OA provided in Swedish primary care by physiotherapists on the metabolic health of individuals with diabetes and OA. Hence, the register-based study compared the metabolic health profiles of those with diabetes and OA who participated in this exercise intervention in Swedish primary care with those with diabetes and OA who did not participate in the same programme. The metabolic health outcomes included BP, HbA1c, HDL, cholesterol levels and body weight.

## Methods

### Study design

This longitudinal register-based study collected prospective healthcare data from the Swedish Osteoarthritis and Diabetes (SOAD) cohort.^19^ The research was conducted with respect to the Declaration of Helsinki and reported following the ‘Strengthening the Reporting of Observational Studies in Epidemiology’.^20^

### Data sources

SOAD incorporates individual-level data from various sources, including the Swedish Osteoarthritis Register (SOAR)^21^ and the National Diabetes Register (NDR). Additional variables regarding individuals’ diagnosis and use of prescribed drugs were obtained through the Swedish Prescribed Drug Register and the National Patient Register. All registries were merged using personal identity numbers unique to all citizens in Sweden.^22^

The SOAR started in 2008 and currently includes more than 230,000 individuals with OA who have sought treatment for OA in Sweden and were referred for standardised core intervention (education and supervised exercises). Currently, SOAR collects data from over 800 physiotherapy units in primary healthcare across Sweden. It has an 86% coverage rate in the Swedish rehabilitation unit and a 72% completeness rate by participants.^23^ Participants started this treatment after a confirmed clinical or radiographic OA diagnosis following the recommendations for OA diagnosis from the Swedish National Board of Health and Welfare.^24^ People could not attend this intervention if they had joint pain in the index joint caused by another disease (eg, sequelae of hip fracture, inflammatory joint disease, cancer) or did not understand Swedish (up to 2016). The intervention targets one index joint, selected by the physiotherapist during the baseline visit based on the participant’s medical history, symptoms and clinical examination. The intervention comprises two parts: (1) education and (2) exercise. The former is mandatory, while the latter is optional. The education part is based on three sessions. The first two sessions are compulsory and held by a physiotherapist. The first two sessions highlight the pathophysiology of the disease and the possible treatments to tackle OA symptoms, such as exercise and self-management strategies. The third one is optional and held by a person with OA trained by the European Osteoarthritis Communicator Programme.^21^ It revolves around their lived experience with this condition and non-surgical interventions. The exercise part (optional) starts with an individual session with a physiotherapist to tailor the exercise programme to the participants’ needs and characteristics. Hence, the participant can decide whether to perform the exercise at home or in supervised group sessions with a physiotherapist twice weekly for 6–8 weeks. In this study, we analysed those who underwent the education and the supervised exercise session(s) with a physiotherapist.

The NDR has been a Swedish National Quality Register since 1996 and collects data on clinical characteristics, risk factors, laboratory analyses, complications of diabetes and medications for people 18 years of age or older with a diagnosis of diabetes. In recent years, the NDR has reached a coverage of >80% of all the people with a diagnosis of diabetes in Sweden.^25^

### Study participants

The study cohort comprised all the participants from the SOAR between January 2008 and December 2019 who underwent the supervised exercise intervention in Swedish primary care for knee or hip OA and who were included in the NDR (have diabetes). We excluded SOAR participants who performed home exercises due to the lack of measures for exercise adherence. This cohort was matched to a control group of individuals in the NDR diagnosed with OA in specialist care who did not undergo the intervention recorded in SOAR. Controls were matched based on age, assigned sex at birth, educational attainment and date of OA diagnosis (exact matching). The index date was the day of enrolment in the intervention for cases and the exact date for matched controls. For each outcome, we included people with at least one available assessment in that specific outcome during this study’s observation period.

### Variables

#### Exposure variable

Having attended the OA intervention recorded in the SOAR was the exposure variable. An interaction between exposure and time was included in the model to consider the effect of exposure as time-varying.

#### Outcome variables

The outcomes of interest were systolic BP (mm Hg), HbA1c (mmol/mol), HDL (mmol/L), total cholesterol levels (mmol/L) and body weight (kg).

### Data analysis

Observations spanned 3 years before and 3 years following the index date, divided into 6-month intervals, with the 6 months preceding the enrolment of a baseline reference. Averages per 6-month intervals were computed since systolic BP, HbA1c, HDL, weight and total cholesterol levels were measured multiple times within the NDR register. A two-way fixed-effect model was used to capture dynamic effects while accounting for time-invariant confounding factors such as educational attainment, sex assigned at birth and genetic background. The impact of participating in the SOAR was treated as time-varying thanks to the interaction between the time variable and the exposure variable in the model. This interaction variable provides an average effect of SOAR enrolment on the observed changes, if any, in the outcomes of interest among the SOAR participants at different time points. With this analysis, given a constant difference in the outcome measurement between the groups before the index date, changes in the differences after the index date can be attributed to the intervention.

Consequently, the difference in the outcomes between cases and controls in the 6 months preceding the index date was normalised to 0. This consideration implies that our estimates can be interpreted as difference-in-difference, that is, the difference between cases and controls at each time point in relation to their difference in the period before the index date. For systolic BP, HbA1c, total cholesterol levels and weight, negative values after the index date would indicate a decrease in the outcome for the cases that can be interpreted as a positive effect of the intervention. For HDL, positive values after the index date would indicate an increase in the outcome for the cases, which can be interpreted as a positive effect of the intervention. The estimated absolute mean values for the exposed and non-exposed groups and the estimated differences-in-difference between the exposed and non-exposed groups were reported. The estimates were reported for systolic BP, HbA1c, HDL, weight and total cholesterol levels at each time point before and after the index date, alongside their 95% CIs. A sensitivity analysis for each outcome was conducted to address the potential bias introduced by having individuals without an assessment on pre-index and post-index dates, including individuals with at least one evaluation before and after the index date. All statistical analyses were performed using Stata V.18 software (StataCorp 2023 Stata Statistical Software: Release 18 College Station, Texas, USA: StataCorp).

## Results

From the SOAR, 4571 individuals with an OA diagnosis in specialist care and diabetes were identified, with a mean age of 69.5 (SD: 7.8) and comprising 52.7% women. Among them, 71.2% had knee OA, and 28.8% had hip OA. Additionally, 7925 matched controls were identified. Table 1 illustrates the descriptive data of both cases and controls. Online supplemental table 1 reports the number of observations at each time point for each outcome variable for cases and controls.

**Table 1:** Descriptive data at the index date

	Swedish Osteoarthritis Registry	Matched controls
N	4571	7925
Age, mean (SD)	69.5 (7.8)	69.6 (7.7)
Female, n (%)	2408 (52.7)	4140 (52.2)
Knee OA, n (%)	3255 (71.2)	5604 (70.7)
Marital status, n (%)		
Never married	516 (11.3)	896 (11.3)
Previously married	1378 (30.2)	2531 (31.9)
Married	2673 (58.5)	4314 (54.4)
Missing	4 (0.1)	184 (2.3)
Educational attainment, n (%)		
0–9 years of education	1411 (30.9)	2696 (34.0)
10–12 years of education	2198 (48.1)	3402 (42.9)
≥13 years of education	952 (20.8)	1519 (19.2)
Missing	10 (0.2)	308 (3.9)
Income quartile*, n (%)		
Lowest quartile	964 (21.1)	2118 (26.7)
Quartile 2	1105 (24.2)	1969 (24.9)
Quartile 3	1291 (28.2)	1785 (22.5)
Highest quartile	1207 (26.4)	1869 (23.6)
Missing	4 (0.1)	184 (2.3)

* People were stratified in different quartiles based on their income.

N, number; OA, osteoarthritisSDStandard Deviation

**Figure 1:**
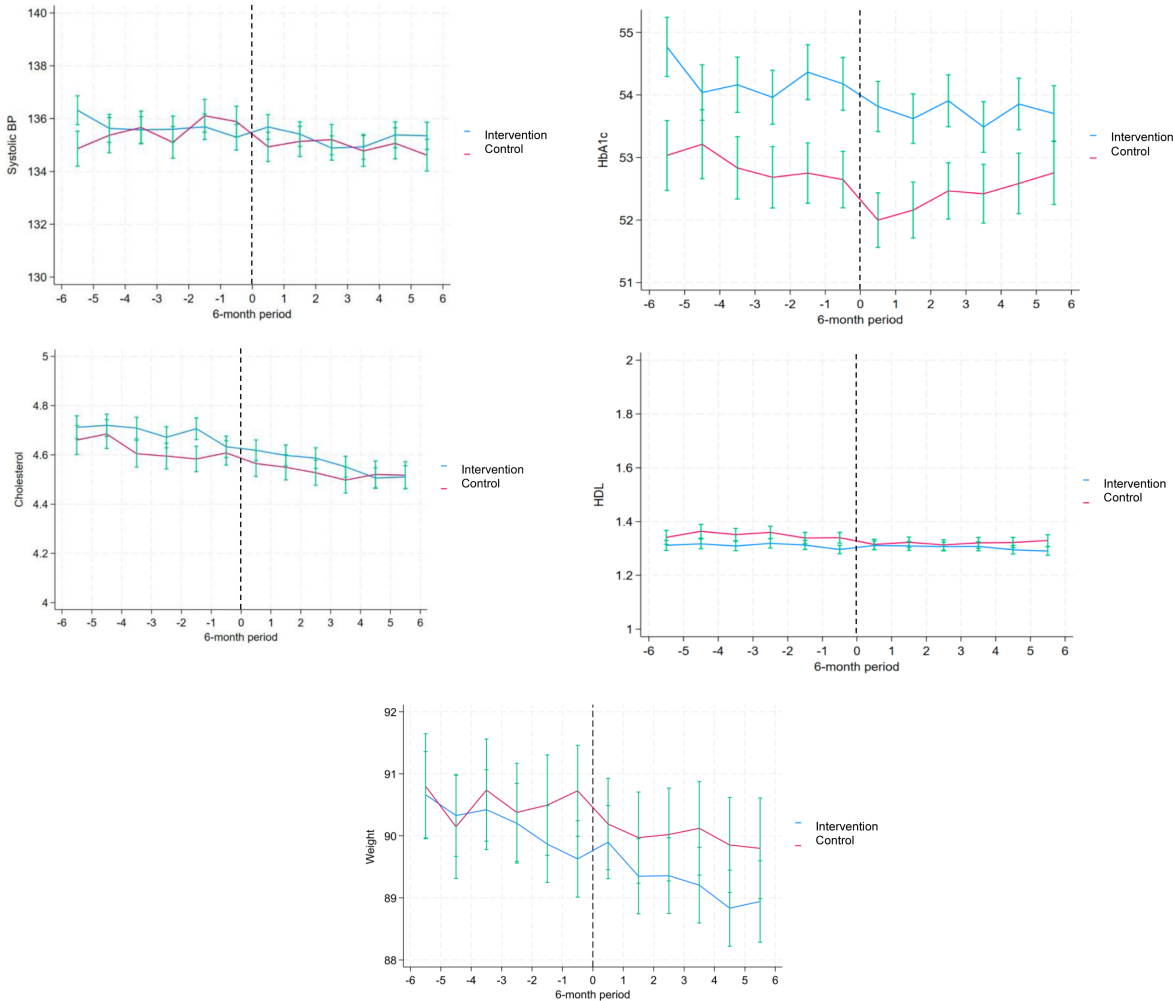
Variations in metabolic health outcomes before and after the intervention. Note: Systolic BP values are reported with mm Hg as unit of measure; HbA1c with mmol/mol; total cholesterol levels and HDL with mmol/L; weight with kg. BP, blood pressure; HbA1c, glycated haemoglobin; HDL, high-density lipoprotein.

**Figure 2:**
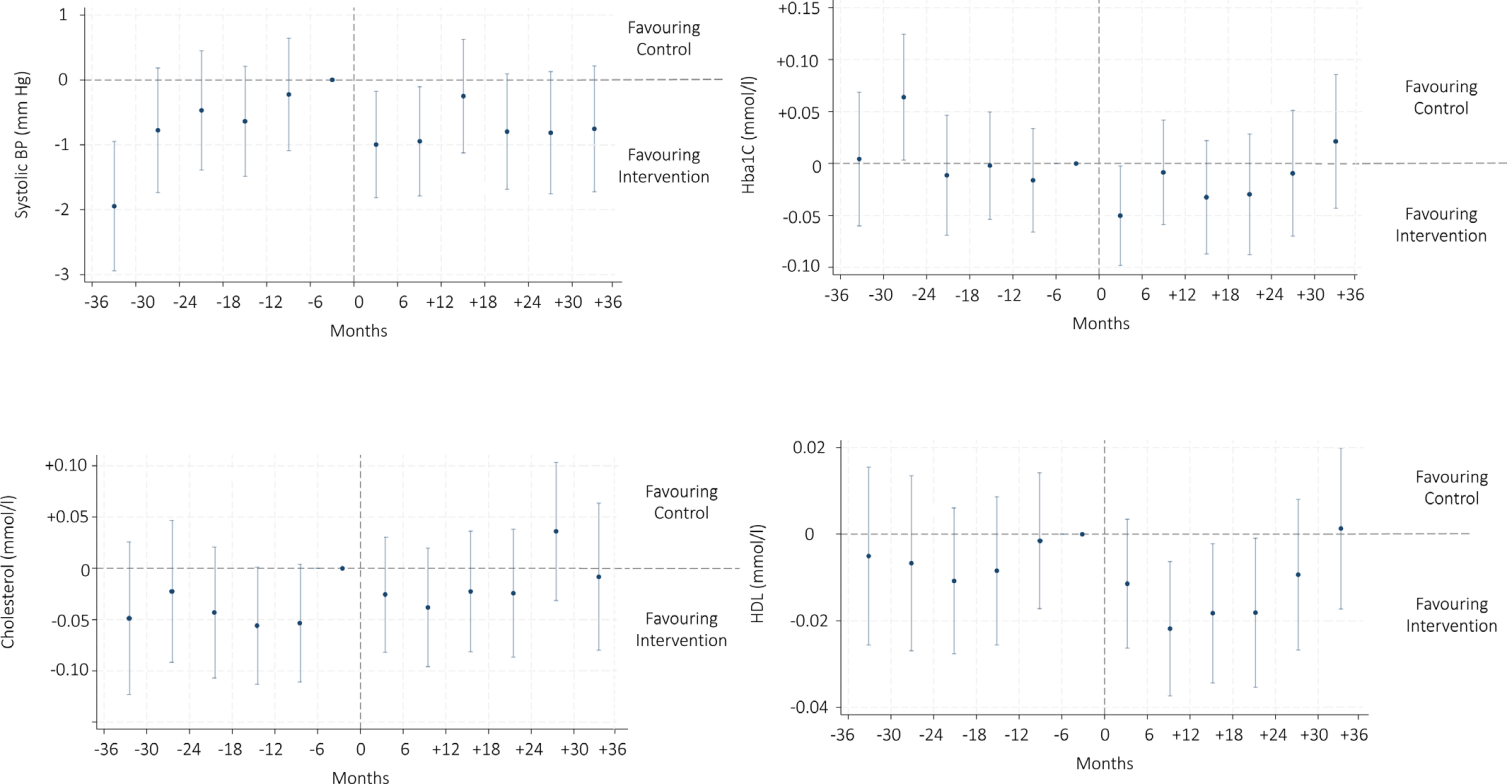
Difference-in-difference estimates in metabolic health outcomes before and after the exercise intervention. BP, blood pressure; HbA1c, glycated haemoglobin; HDL, high-density lipoprotein.

Cholesterol, HbA1c and weight showed a decreasing trend during the observational period in both cases and controls, while systolic BP and HDL appeared overall stable (figure 1). These estimates provide insight into the average association of attending the first-line intervention for OA recorded in SOAR on the metabolic outcomes among intervention participants. When studying the average association of participating in the intervention on the metabolic outcomes, we observed a decrease of 1.0 mm Hg (95% CI (−1.80; −0.20)) in systolic BP at 6 months post-intervention, which persisted at 12 months, (−1.0 mm Hg (−1.80; −0.10)) (figure 2, online supplemental table 2). HbA1c levels decreased at the 6-month follow-up (−0.50 mmol/L (−0.98; −0.023)). HDL levels decreased at the 12-month (−0.02 mmol/L (−0.04; −0.01)), 18-month (−0.02 mmol/L (−0.04; −0.01)) and 24-month (−0.02 mmol/L (−0.03; −0.01)) measurements. Weight decreased at 6 months (−0.42 kg (−0.71; −0.13)) and 18 months (−0.50 kg (−0.84; −0.15)), persisting at 24 months (−0.42 kg (−0.80; −0.04)) and 30 months (−0.42 kg (−0.80; −0.04)). No association with intervention was observed in total cholesterol levels over time.

Weight was the only outcome to show unstable values before the index date, meaning that it is uncertain whether subsequent differences estimated by our model reflect the actual association with the intervention. For all the other outcomes, differences before the index date were stable around 0 (no difference). The sensitivity analysis with only individuals with at least one assessment before and one after the index date confirmed the results of the main analysis (online supplemental table 3).

## Discussion

Based on the study’s findings, our results suggested that people with diabetes and OA who participated in a self-management 6-week education and exercise intervention for OA had, on average, slightly improved metabolic health compared with the matched-control group. However, the observed average differences between the two groups appear small in magnitude and unlikely to be clinically relevant.

The observed small treatment effects raise essential questions regarding the effectiveness of OA self-management interventions designed following clinical practice guidelines in improving metabolic health in individuals with comorbid diabetes. Considering recent evidence suggesting that the efficacy of exercise in reducing joint pain may be lower than previously thought,^26^ our results raise further questions on the effectiveness of OA first-line management in those with OA and comorbid metabolic health problems. Specifically, we have not observed essential improvements in our cohort’s metabolic profile when compared with a matched control group. Moreover, for HbA1c levels and weight, the differences could be due to normal within-subject variability of the measurement.^27^ Clinically significant differences for metabolic parameters would be considered as a 4 mm Hg reduction for systolic BP,^28^ 5 mmol/mol for HbA1c, 5% for body weight,^29^ 0.26 mmol/L for total cholesterol level and an improvement of 0.10 mmol/L for HDL.^30^

One possible explanation for the limited efficacy of the analysed intervention could be the programme’s short duration in achieving behavioural and metabolic changes. Previous evidence suggested that most health-related variables reversed quickly following a month of detraining.^31^ It is unknown if this programme made long-lasting changes in the participant’s physical activity levels. However, it is essential to note that this intervention aims to teach how to manage OA, not metabolic health. Moreover, in people with OA, it is well-known that long-term adherence to an exercise protocol can be low.^17^ Exercise can trigger a synchronised interaction among various tissues to meet the heightened energy requirements^32^ and, over time, drive enduring adaptations across multiple tissues, encompassing the cardiovascular system, skeletal muscle, adipose tissue, liver, pancreas, gut and brain.^32^ However, how long it takes to reach metabolic changes has yet to be understood, though at least 12 weeks of training (twice the length of this programme) seemed necessary to get small beneficial changes.^33^

As per the intensity, a recent systematic review highlighted that the recommended optimal dose of physical activity identified for glucose control in type-2 diabetes is 1100 metabolic equivalent of task-min/week (METs-min/week), which corresponds to 244 min per week of moderate-intensity aerobic activity or 157 min per week of vigorous-intensity aerobic activity.^34^ These parameters exceed the current recommendations for physical activity in the general population, in people with diabetes, people with OA^35^ and the provided training intensity of the present programme, which consists of two 60 min sessions of exercise per week for 6 weeks.^36^ Therefore, future studies should investigate whether the physical activity and exercise recommendation for OA and diabetes should be adapted for people with both conditions to achieve an appropriate dose that can improve metabolic health while accounting for the limitations imposed by OA. Additionally, research should explore the effects of long-term interventions with intensified exercise protocols explicitly tailored for individuals with diabetes and OA.

This calls for a potential reassessment of our current intervention strategies to enhance OA management by critically evaluating the adopted approach. Although the average benefits appear minimal compared with controls, this could indicate that current interventions are on the right path and that some individuals may benefit. OA management should account for overall health, and better-tailored exercise interventions may need to be adapted to the comorbidity profiles of people, indicating that a one-size-fits-all approach may be insufficient to address the complex interplay between metabolic health and OA.^4^ Therefore, our findings suggest that clinicians should consider comorbid metabolic conditions when prescribing exercise for individuals with OA, using metabolic health changes as objective measures of exercise success.

Several limitations of this study need to be acknowledged. First, we need to highlight that the primary aim of the intervention recorded in the SOAR was not focused on metabolic health but on how to self-manage OA and exercise while having this condition (without a focus on comorbidities such as diabetes). Second, we reported average changes in the outcomes, which do not exclude that specific subgroups of individuals may have experienced clinically relevant benefits. Additionally, the benefits might not be solely credited to the exercise sessions. Self-management programmes aim to help individuals take proactive steps in managing their conditions. Thus, individuals participating in the intervention may have been empowered to self-manage their joint symptoms and may have resorted to additional care. Similarly, individuals in the control group may have resorted to additional care that could not be captured through the registers. The study’s observational nature precludes establishing causality and concluding the relationship between this intervention and metabolic outcomes. Therefore, our results should be further confirmed on other populations and through randomised controlled trials (RCTs). Another potential limitation is the timing at which the outcomes were measured. We observed that some participants had numerous measurements, likely those with poorer metabolic control. In contrast, others had no measurements within the first 6 months after the intervention, a period where effects are more likely to be observed. Furthermore, the study primarily included individuals with an OA diagnosis from specialist care. This could be a limitation if people with OA who have primary care contact generally have less severe disease and are thus able to exercise ‘more’ or ‘better’, potentially gaining more benefit from the intervention, as shown in a digital self-management programme of this intervention.^37^ Despite these limitations, it is noteworthy that the current study included data from over 10 000 individuals with OA and diabetes sourced from national Swedish registries. Our study’s substantial size and high-quality data enhance its relevance for further research.

In conclusion, our study suggested that an OA first-line management programme that provides exercise and education for 6 weeks, according to available clinical practice guidelines, offered a slight benefit to the metabolic health of individuals with OA and comorbid diabetes. Still, the differences were, on average, small and of unclear clinical significance. This study underscores the importance of advancing exercise interventions tailored to the unique needs of individuals with diabetes and OA. Future research should create OA exercise training that optimises exercise parameters for people with OA and diabetes (eg, intensity, duration, dosage) and include objective measures (eg, MET, heart rate and oxygen consumption peak). By integrating innovative exercise strategies and considering individual variability in treatment response, the management of diabetes and OA can potentially alleviate the burden associated with these long-term conditions.

## supplementary material

10.1136/rmdopen-2024-005133 online supplemental table 1

10.1136/rmdopen-2024-005133 online supplemental table 2

10.1136/rmdopen-2024-005133 online supplemental table 3

## Data Availability

Data are available upon reasonable request.
