# Supplementary material for: Impact of an intervention for osteoarthritis based on exercise and education on metabolic health: a register-based study using the SOAD cohort
Source: RMD Open. 2025 Feb 26;11(1):e005133. doi: 10.1136/rmdopen-2024-005133 (PMC11865791; doi:10.1136/rmdopen-2024-005133)
Supplement: online supplemental table 1 [file rmdopen-11-1-s001.docx]

**Supplementary Table 1** Number of observations per each time point

| Time Periods | Systolic Bp  N | | Hba1c  N | | HDL  N | | Cholesterol  N | | Weight  N | |
| --- | --- | --- | --- | --- | --- | --- | --- | --- | --- | --- |
|  | **SOAR** | **Control** | **SOAR** | **Control** | **SOAR** | **Control** | **SOAR** | **Control** | **SOAR** | **Control** |
| -30 Months | 1,705 | 2,893 | 1,733 | 2,921 | 1,088 | 1,951 | 1,245 | 2,149 | 1,561 | 2,578 |
| -24 Months | 1,778 | 2,994 | 1,811 | 3,078 | 1,119 | 2,002 | 1,276 | 2,216 | 1,626 | 2,713 |
| -18 Months | 1,943 | 3,251 | 1,983 | 3,305 | 1,242 | 2,159 | 1,412 | 2,371 | 1,797 | 2,898 |
| -12 Months | 2,065 | 3,372 | 2,119 | 3,421 | 1,369 | 2,227 | 1,532 | 2,410 | 1,880 | 2,959 |
| -6 Months | 2,134 | 3,536 | 2,195 | 3,582 | 1,404 | 2,336 | 1,557 | 2,535 | 1,928 | 3,095 |
| Baseline | 2,414 | 3,659 | 2,477 | 3,720 | 1,598 | 2,398 | 1,761 | 2,590 | 2,177 | 3,154 |
| 6 Months | 2,361 | 3,861 | 2,438 | 3,910 | 1,528 | 2,577 | 1,677 | 2,765 | 2,139 | 3,406 |
| 12 Months | 2,410 | 3,863 | 2,494 | 3,957 | 1,616 | 2,566 | 1,761 | 2,749 | 2,141 | 3,389 |
| 18 Months | 2,443 | 3,821 | 2,512 | 3,936 | 1,587 | 2,590 | 1,731 | 2,751 | 2,166 | 3,359 |
| 24 Months | 2,300 | 3,728 | 2,395 | 3,829 | 1,531 | 2,534 | 1,650 | 2,695 | 2,053 | 3,233 |
| 30 Months | 2,244 | 3,570 | 2,322 | 3,660 | 1,529 | 2,430 | 1,646 | 2,581 | 1,973 | 3,148 |
| 36 Months | 2,072 | 3,241 | 2,164 | 3,323 | 1,410 | 2,207 | 1,493 | 2,335 | 1,826 | 2,794 |

**Legend:** BP, blood pressure; HbA1c: Glycated Haemoglobin; HDL, High-density lipoprotein; N, number; SOAR, Swedish Osteoarthritis Registry.
