# Supplementary material for: Impact of an intervention for osteoarthritis based on exercise and education on metabolic health: a register-based study using the SOAD cohort
Source: RMD Open. 2025 Feb 26;11(1):e005133. doi: 10.1136/rmdopen-2024-005133 (PMC11865791; doi:10.1136/rmdopen-2024-005133)
Supplement: online supplemental table 2 [file rmdopen-11-1-s002.docx]

**Supplementary Table 2** Variations in Metabolic Health Outcomes before and after the Intervention

|  | **Systolic BP (mmHg)** | **HbA1c (mmol/mol)** | **HDL (mmol/l)** | **Cholesterol (mmlo/l)** | **Weight (kg)** |
| --- | --- | --- | --- | --- | --- |
| **Time periods** | Mean Difference [95% Confidence Interval] | | | | |
| **-30 months** | -2.0 [-2.94; -0.95] | 0.0 [-0.60; 0.69] | 0.0 [-0.03; 0.02] | -0.1 [-0.12; 0.03] | -0.7 [-1.10; -0.31] |
| **-24 months** | -0.8 [-1.74; 0.18] | 0.6 [0.03; 1.25] | 0.0 [-0.03; 0.01] | 0.0 [-0.09; 0.05] | -0.6 [-0.96; -0.23] |
| **-18 months** | -0.5 [-1.39; 0.45] | -0.1 [-0.69; 0.46] | 0.0 [-0.03; 0.01] | 0.0 [-0.11; 0.02] | -0.4 [-0.76; 0.00] |
| **-12 months** | -0.6 [-1.48; 0.21] | 0.0 [-0.54; 0.50] | 0.0 [-0.03; 0.01] | -0.1 [-0.11; 0.00] | -0.6 [-0.91; -0.30] |
| **-6 months** | -0.2 [-1.09; 0.64] | -0.2 [-0.66; 0.34] | 0.0 [-0.02; 0.01] | -0.1 [-0.11; 0.00] | -0.3 [-0.61; 0.06] |
| **Baseline** | 0 | 0 | 0 | 0 | 0 |
| **6 months** | -1.0 [-1.82; -0.18] | -0.5 [-0.98; -0.03] | 0.0 [-0.03; 0.00] | 0.0 [-0.08; 0.03] | -0.4 [-0.71; -0.13] |
| **12 months** | -1.0 [-1.79; -0.11] | -0.1 [-0.59; 0.42] | 0.0 [-0.04; -0.01] | 0.0 [-0.10; 0.02] | -0.2 [-0.51; 0.11] |
| **18 months** | -0.3 [-1.13; 0.62] | -0.3 [-0.87; 0.22] | 0.0 [-0.03; 0.00] | 0.0 [-0.08; 0.04] | -0.5 [-0.84; -0.15] |
| **24 months** | -0.8 [-1.68; 0.09] | -0.3 [-0.88; 0.29] | 0.0 [-0.04; 0.00] | 0.0 [-0.09; 0.04] | -0.4 [-0.79; -0.04] |
| **30 months** | -0.8 [-1.76; 0.13] | -0.1 [-0.70; 0.51] | 0.0 [-0.03; 0.01] | 0.0 [-0.03; 0.10] | -0.4 [-0.79; -0.04] |
| **36 months** | -0.8 [-1.72; 0.22] | 0.2 [-0.43; 0.86] | 0.0 [-0.02; 0.02] | 0.0 [-0.08; 0.06] | -0.4 [-0.84; 0.04] |

Legend: BP, blood pressure; HDL, High-density lipoprotein.
