# Supplementary material for: Impact of an intervention for osteoarthritis based on exercise and education on metabolic health: a register-based study using the SOAD cohort
Source: RMD Open. 2025 Feb 26;11(1):e005133. doi: 10.1136/rmdopen-2024-005133 (PMC11865791; doi:10.1136/rmdopen-2024-005133)
Supplement: online supplemental table 3 [file rmdopen-11-1-s003.docx]

**Supplementary Table 3** Sensitivity analysis - Differences in Metabolic Health Outcomes before and after the Intervention when including only people with at least one assessment before and one after the inclusion in the SOAR

|  | **Systolic BP (mm Hg)** | **HbA1c (mmol/mol)** | **HDL (mmol/l)** | **Cholesterol (mmlo/l)** | **Weight (kg)** |
| --- | --- | --- | --- | --- | --- |
| **Time period** | Mean Difference [95% Confidence Interval] | | | | |
| **-30 months** | -2.0 [-3.01; -1.00] | 0.1 [-0.54; 0.77] | 0.0 [-0.03; 0.02] | -0.1 [-0.12; 0.03] | -0.7 [-1.11; -0.30] |
| **-24 months** | -0.7 [-1.68; 0.26] | 0.7 [0.05; 1.29] | 0.0 [-0.03; 0.02] | 0.0 [-0.09; 0.05] | -0.6 [-1.02; -0.27] |
| **-18 months** | -0.5 [-1.46; 0.40] | -0.2 [-0.75; 0.43] | 0.0 [-0.03; 0.01] | 0.0 [-0.11; 0.02] | -0.4 [-0.78; -0.01] |
| **-12 months** | -0.7 [-1.58; 0.14] | 0.0 [-0.55; 0.51] | 0.0 [-0.03; 0.01] | -0.1 [-0.12; 0.00] | -0.7 [-0.96; -0.34] |
| **-6 months** | -0.2 [-1.09; 0.68] | -0.2 [-0.69; 0.33] | 0.0 [-0.02; 0.01] | -0.1 [-0.11; 0.01] | -0.3 [-0.65; 0.03] |
| **Baseline** | 0 | 0 | 0 | 0 | 0 |
| **6 months** | -1.0 [-1.84; -0.18] | -0.4 [-0.88; 0.06] | 0.0 [-0.03; 0.00] | 0.0 [-0.08; 0.03] | -0.4 [-0.73; -0.15] |
| **12 months** | -0.9 [-1.73; -0.01] | -0.2 [-0.67; 0.35] | 0.0 [-0.04; 0.00] | -0.1 [-0.11; 0.01] | -0.2 [-0.56; 0.07] |
| **18 months** | -0.3 [-1.16; 0.64] | -0.3 [-0.82; 0.30] | 0.0 [-0.03; 0.00] | 0.0 [-0.08; 0.04] | -0.6 [-0.92; -0.20] |
| **24 months** | -0.8 [-1.75; 0.09] | -0.4 [-1.01; 0.18] | 0.0 [-0.04; 0.00] | 0.0 [-0.08; 0.05] | -0.5 [-0.88; -0.09] |
| **30 months** | -1.0 [-2.02; -0.06] | 0.0 [-0.60; 0.66] | 0.0 [-0.02; 0.01] | 0.1 [-0.01; 0.13] | -0.4 [-0.78; -0.02] |
| **36 months** | -0.7 [-1.69; 0.34] | 0.1 [-0.56; 0.78] | 0.0 [-0.02; 0.02] | 0.0 [-0.09; 0.06] | -0.3 [-0.80; 0.13] |

Note: Only people with at least one assessment before and one after the inclusion in the SOAR are included in this sensitivity analysis
